# Morphologic and histologic characterization of sheep and porcine TMJ as large animal models for tissue engineering applications

**DOI:** 10.1007/s00784-022-04472-3

**Published:** 2022-04-01

**Authors:** Jonah D. Lee, Josh I. Becker, Lisa M. Larkin, Alejandro J. Almarza, Sunil D. Kapila

**Affiliations:** 1 Department of Molecular & Integrative Physiology, University of Michigan School of Medicine, Ann Arbor, MI 48109 USA; 2 Animal Care and Use Office, Office of Research, University of Michigan, Ann Arbor, MI 48109 USA; 3 Department of Oral and Craniofacial Sciences, School of Dental Medicine, University of Pittsburgh, Pittsburgh, PA 15261 USA; 4 Center of Craniofacial Regeneration, University of Pittsburgh, Pittsburgh, PA 15261 USA; 5 Department of Bioengineering, Swanson School of Engineering, University of Pittsburgh, Pittsburgh, PA 15261 USA; 6 Section of Orthodontics, School of Dentistry, University of California Los Angeles School of Dentistry, 10833 Le Conte Avenue, CHS 33-089, Box 951668, Los Angeles, CA 90095 USA

**Keywords:** Temporomandibular joint, Sheep, Pig, Tissue engineering, Micro-CT

## Abstract

**Objective:**

The aim of this study was to compare and characterize the structural and ultrastructural organization of the temporomandibular joint (TMJ) between two large animal models for use in the development of tissue engineering strategies.

**Materials and methods:**

Whole TMJs from sheep and pigs were evaluated with micro-computed tomography (μCT) for morphology and quantitative analyses of bone parameters. Histological examination was performed on the TMJ disc and its attachments to investigate regional distribution of collagen, elastin, and glycosaminoglycans (GAGs).

**Results:**

μCT analyses demonstrate higher bone mineral density (BMD) in the temporal fossa compared to the mandibular condyle in both species, with this variable being significantly higher in sheep than pig. Quantitative morphometry of the trabecular condyle reveals no statistical differences between the species. Histology demonstrates similar structural organization of collagen and elastin between species. Elastin staining was nearly twofold greater in sheep than in the pig disc. Finally, Safranin-O staining for GAGs in the TMJ disc was localized to the intermediate zone in the sheep but was absent from the porcine disc.

**Conclusions:**

Our findings show some important differences in the pig and sheep TMJ μCT variables and histology and composition of the disc and discal attachment. These disparities likely reflect differences in masticatory and TMJ functional loading patterns between the two species and provide insights into large animal models towards human applications.

**Clinical relevance:**

As with the established pig model, the sheep is a suitable large animal model for TMJ research such as regenerative strategies, with specific considerations for design parameters appropriate for human-analog applications.

## Introduction

Patients that present with temporomandibular joint (TMJ) dysfunction, inflammation, pain, and degeneration, suffer from degenerative joint disease (DJD). Because the causes of TMJ DJDs are largely unknown and due to the complexity and uniqueness of this joint, management of TMJ diseases is conservative and non-specific, and the regeneration of joint structures remains perplexing and underexplored [1–3]. The lack of consensus on proven treatment strategies for TMJ DJDs further emphasizes a need for more comprehensive studies in the TMJ, and the need regenerative strategies specifically targeted to the TMJ.

The TMJ has several unique characteristics, such as its ability to rotate and translate as a ginglymoarthrodial joint. Furthermore, these bilateral joints need to function in unison during essential activities including mastication, speech, respiration, and swallowing. Additionally, the TMJ has many connective tissues, with articular surfaces comprised of fibrocartilage, and an intervening fibrocartilaginous disc. Lastly, the developmental origins of the TMJ are from neural crest cells. These unique attributes of the TMJ are unlike those most other joints, which are comprised of hyaline articular cartilage derived from mesoderm, and that work only in rotation. Thus, regenerative therapies in the knee or other similar joints cannot be readily translatable to the TMJ [4] highlighting the need for discoveries and approaches for therapies that are customized to the TMJ.

A major obstacle in pursing regenerative strategies of the TMJ is a dearth of comparative characterization evaluating suitable experimental animal models, particularly in the large animal, to advance technologies from benchtop to bedside [5, 6]. While the pig has often been cited as the gold standard model for human TMJ anatomy, there are aspects that limit the use of the pig for tissue engineering strategies [5]. Specifically, in the pig surgical access to the joint is blocked by the zygomatic arch, and the farm pig has exponential growth for 18 months of age [5], adding a unique developmental variable to anatomical studies. These limitations of the pig model have prompted the investigation of other large animal models, such as the sheep [5]. Indeed, several studies have used the sheep as a model for different TMJ pathologies and surgeries [7–11]. Nevertheless, adequate characterization of the sheep TMJ and its comparison with that of the pig are largely lacking. The aim of this study was to directly contrast the morphological and histological differences between two large animal models utilized for TMJ research for the purposes of characterizing structural organization and biochemical content of the disc and discal attachments, and bone morphology. The findings of this study will help to identify and associate unique morphological and functional characteristics of large animal TMJs for future therapeutic strategies of orofacial regenerative medicine.

## Materials and methods

### Specimen preparation

TMJs were excised *en bloc* from slaughter-aged adult animals: 5 sheep of approximately 10–13 months of age (Suffolk or Dorset; Valley View Farms, Cockeysville, MD or Cornell Farms, Dryden, NY) and 5 pigs of approximately 5–7 months of age (Yorkshire or Landrace; Whiteshire Hamroc Farms, Albion, IN; Michael Fanning Farms Howe, IN; or Sioux-Preme Products, Sioux Center, IA). One adult male human sample obtained from the University of Michigan Anatomical donation program in accordance with provisions from the Michigan anatomical gift law (Public Act #368 of 1978, amended Public Act #39 of 2008; Medical School, University of Michigan, Ann Arbor, MI) was used for comparison of morphologic and micro-computed tomography (μCT) findings. For each animal sample, one joint was used for μCT and the other joint for histology. For μCT analysis, dissection of the joint was performed carefully with a high-performance oscillating electric saw to maintain an intact join capsule and include portions of the temporal bone and the condyle. Segments of the zygomatic process, squama temporalis, mastoid portion were dissected to maintain the integrity of the joint during removal. A condylectomy was performed and the joint capsule removed *en bloc*, washed with phosphate-buffered saline (PBS, Sigma-Aldrich, St. Louis, MO, USA), wrapped in gauze soaked with PBS-containing protease inhibitors (1 mM N-ethylmaleimide and 1 mM phenylmethylsulfonyl fluoride, Sigma-Aldrich), and frozen at − 20** °C** until μCT scanning and subsequent sample analyses. For histological analyses, the TMJ disc-attachment complex was isolated and released from the joint capsule temporal and condylar attachments and verified to be grossly normal by visualizing the absence of perforation or deformity and then flash frozen for subsequent histological analyses.

### Micro-computed tomography

Whole joints were evaluated with micro-computed tomography (μCT) for morphology and bone variables: bone mineral density (BMD), bone volume fraction (BVF; percent of the total ROI that is occupied by mineralized bone tissue), porosity (1-BVF; volume fraction of bone not occupied by bone tissue), specific bone surface (BS/BV; ratio of bone surface for a given volume of bone), trabecular bone thickness (TbTh; the average thickness of the trabeculae), trabecular number (TbN; average number of trabeculae per unit length), and trabecular spacing (TbS; mean distance between trabeculae). μCT was performed using eXplore Locus RS (GE Healthcare Pre-Clinical Imaging, London, Ontario, Canada), with the following parameters: voxel size of 46 μm, voltage of 80 kV, current of 450 μA, and an exposure time of 18 min. A hydroxyapatite phantom was used in each scan to calibrate to Hounsfield units for densitometry. Regions of interest (ROIs) were selected by using a spline function to manually contour a region encompassing the surface and subchondral region of the condyle and the corresponding articulating fossa directly superior. ROI placement included the center of the condyle for trabecular bone analyses. The condyle surface ROIs and articulating fossa ROIs were assessed using densitometric analysis (BMD). The trabecular ROIs were assessed both with densitometry and morphology analyses to obtain quantitative outcomes [12]. Three-dimensional (3D) images of the pig and sheep TMJs were constructed for purposes of gross morphologic comparisons.

### Histochemical and immunohistochemical analysis

Frozen samples of TMJ disc and discal attachments were cryo-sectioned at 12 μm thickness from the midline in the antero-posterior direction, mounted on Superfrost Plus microscopy slides, and stained for histological analysis. Tissue sections were analyzed in a region-specific manner that included the anatomical regions of the TMJ disc complex including the interface discal attachments: posterior (P), posterior-intermediate (PI), intermediate articular disc (I), anterior-intermediate (AI), and anterior (A) regions. Sections were stained with hematoxylin and eosin (H&E) for general morphology, and with collagen type I and elastin antibodies, and with Safranin-O for regional content detection of collagen, elastin, and glycosaminoglycans (GAGs), respectively. Sections were fixed with ice-cold methanol for 10 min and rinsed with Dulbecco’s PBS (DPBS). The sections were submerged for 15 min in PBS with 0.05% Triton X-100 (PBST, Sigma-Aldrich) and blocked with containing 3% bovine serum albumin (BSA; Sigma-Aldrich, A2153-10 g) in PBST at room temperature. The sections were then incubated overnight at 4** °C** with primary antibodies to collagen type I (Abcam, Cambridge, MA, USA, #AB292) or elastin (Millipore, Billerica, MA, USA, #AB2039) diluted in PBST containing BSA. Following three PBST washes, sections were incubated in 1:500 dilutions of Alexa-fluor anti-mouse or anti-rabbit antibodies (Life Technologies, Carlsbad, CA, USA) for 3 h at room temperature. Sections were imaged with an Olympus BX-51 microscope and cross-sections were analyzed using Image J software package.

### Statistical analysis

Values are presented as means ± S.E. Measurements of significant differences between means were performed using JMP statistical analysis software (JMP, Cary, NC, USA). Comparisons were made using a one-way analysis of variance with Tukey post hoc analysis when indicated, or paired Student’s *t*-test. Differences were considered significant at *p* ≤ 0.05.

## Results

### Micro-computed tomography (μCT)

**Fig. 1:**
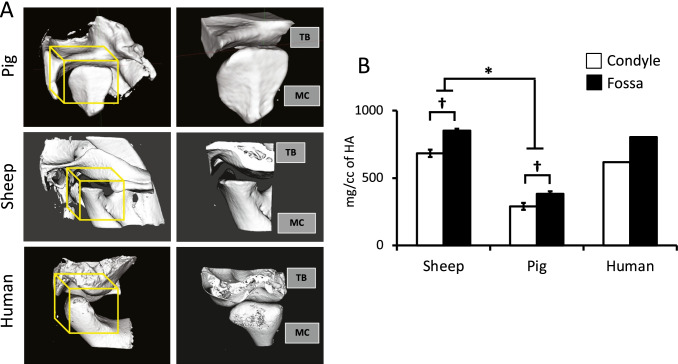
Species-specific morphology and bone mineral density (BMD) of pig (*n* = 5), sheep (*n* = 5), and human (*n* = 1) temporomandibular joints (TMJs). **A** Sagittal volumetric of micro-computed tomography of the right TMJ in pig, sheep, and human including the temporal bone (TB) and condyle of the mandible (CM) (left panel) and an enlarged cropped image (right panel) to emphasize regions of interest (ROI; yellow cube) that encompass the surface and subchondral region of the condyle and corresponding articulating glenoid fossa directly superior. **B** BMD from ROIs in each species show significantly greater density in the fossa compared to the condyle and more dense bone in sheep than in pigs in both these regions. Data represented as mean ± standard error of the mean (S.E.M.). Significant difference between *species and ^†^ROI in condyle vs. fossa, *p* < 0.05

A three-dimensional reconstruction of the scanned images was utilized to visualize different structures throughout the temporal and mandibular bones and identify ROIs for quantitative analyses (Fig. 1A). In the pig, the mandibular condyle extends in a latero-medial direction. The articular surface of the temporal bone is concave on one side and convex on the other and thickest at the periphery (concavo-convex) and joins the upper articular surface of the meniscal attachments to form a reciprocally fitting disco-temporal joint. The articular surface of the mandibular condyle is convex and joins the lower articular surface of the disc, which is concave, to form a condylo-discal complex. These porcine characteristics are most similar to those of human. In the sheep, notable observed differences from human and pig include the articular surface of the temporal bone which is slightly convex and joins the upper articular surface of the disc, which is slightly concave, whereas the articular surface of the mandibular condyle is concavo-convex and joins the lower articular surface of the disc to form a reciprocally fitting condylo-discal interface. These morphological observations support previously reported findings for the pig model [13] and also highlight the differences in biomechanics of the joints from the two species, emphasizing important considerations relative to the human joint.

Mandibular condyle versus glenoid fossa of the temporal bone evaluation demonstrated a significantly lower BMD in the condyle compared to the fossa in both sheep and pig (Fig. 1B). BMD density was significantly lower in the TMJ joint of the pig compared to that of the sheep (Fig. 1B). As a comparison the BMD of the human condyle (585.7 mg/cc of HA) and fossa (754.0 mg/cc of HA) lies between that of sheep and pig, with a value more comparable to that of sheep. Quantitative morphometry of the trabecular bone in the condyle did not reveal any statistical differences between the pig and sheep in BVF, porosity, BS/BV, TbTh, TbN, and TbS (Table 1). Furthermore, both species seem to be equivalent in these measures to the human condyle.

**Table 1:** Quantitative morphometry of the trabecular condyle architecture for pig (*n* = 5), sheep (*n* = 5), and human (*n* = 1) for bone volume fraction (BVF), porosity (1-BVF), specific bone surface (BS/BV), trabecular bone thickness (TbTh), trabecular number (TbN), and trabecular spacing (TbS). Data represented as mean ± standard error of the mean (S.E.M.)

	BVF (%)	Porosity (%)	BS/BV (mm^2^/mm^3^)	Tb. Th (mm)	Tb. N. (1/mm)	Tb. S. (mm)
Human	36	64	30.6	0.07	5.57	0.12
Pig	49 ± 0	51 ± 0	25.7 ± 1.3	0.08 ± 0.00	6.17 ± 0.35	0.09 ± 0.01
Sheep	51 ± 3	49 ± 3	18.6 ± .08	0.11 ± 0.00	3.58 ± 0.44	0.18 ± 0.03
*p*-value between pig and sheep	0.10	0.48	0.08	0.08	0.08	0.10

### Histochemical and immunohistochemical analysis

**Fig. 2:**
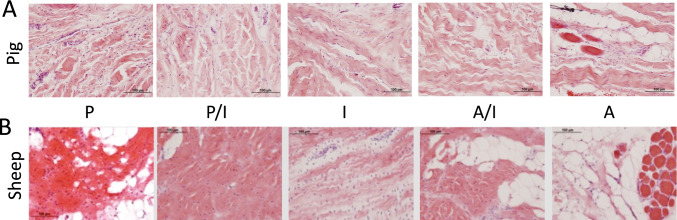
Region-specific cellular and extracellular orientation and organization detected throughout the TMJ disc-ligament-muscle complex with hematoxylin and eosin (H&E) staining in porcine (**A**) and sheep (**B**) TMJ discs. Regions of the TMJ disc-ligament-muscle complex as a whole include posterior (P), posterior-intermediate (PI), Intermediate articular disc (I), anterior-intermediate (AI), and anterior (A) regions. Scale bar = 100 μm

General morphology assessed via H&E staining (Fig. 2) demonstrated a well-defined region-specific orientation throughout the TMJ disc complex that includes the disc and discal attachment interfaces in both the pig and sheep. The TMJ disc is attached along its entire periphery to both the condyle and the temporal bone through a complex network of fibrous connective tissues that form a synovial capsule that envelops the joint [14]. For both the sheep and pig, the posterior of the complex is comprised of a blended network of fibro-elastic tissues and vasculature, which transitions into a more thickly, bundled collagen dense arrangements in the periphery arranged in a circular pattern around the disc (Fig. 2, P and P/I panels). In the intermediate portion of the TMJ fibrocartilage disc, the characteristic crimping patterns of connective tissue become oriented more in antero-posterior in organization, and transitions obliquely once again towards the periphery (Fig. 2, I and A/I panels), establishing the discal, capsular attachments in a circumferential organization. The posterior and anterior regions of the sheep disc (P and A, Fig. 2B) shows more disorganized tissue with greater fat content than similar sites of the pig disc (P and A, Fig. 2A).

**Fig. 3:**
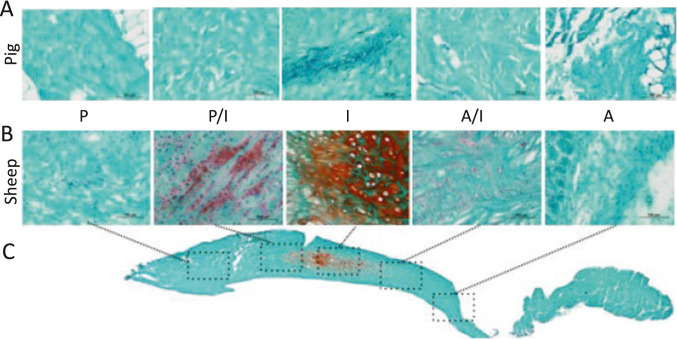
Staining for Safranin-O (red) of the **A** pig and **B** sheep TMJ disc-ligament complex demonstrates the absence of glycosaminoglycan (GAG) staining in the in the pig and staining primarily found in the intermediate (I) discal region of the sheep. **C** GAG staining in the sheep disc visualized at lower magnification composite image. Scale bar = 100 μm

Safranin-O staining was used to localize extracellular matrix (ECM) macromolecules, GAGs that are indicative of compressive properties in the disc fibrocartilage. GAG content was present in the intermediate (I) discal region and posterior-intermediate (P/I) discal attachment region of the sheep TMJ disc, with no other region staining positively for this macromolecule (Fig. 3). Surprisingly, no region of the pig TMJ disc stained positive for GAG.

**Fig. 4:**
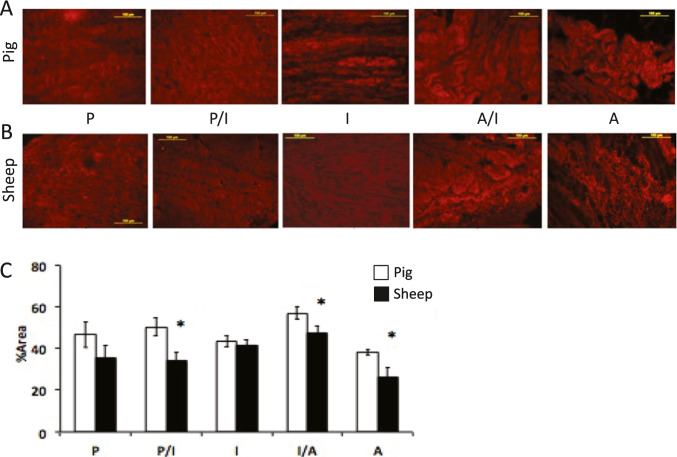
Immunohistochemistry staining for type I collagen of the TMJ disc-ligament complex demonstrates fibrocartilage organization that is region-specific with characteristic crimping fibers of **A** pig and **B** sheep. **C** Quantification of stain intensity for type I collagen content shows a lower percent area in the immediate disc periphery (P/I and I/A) and anterior (**A**) region in the sheep compared to pig. Data represented as mean ± standard error of the mean (S.E.M.). Significant difference between *species, *p* < 0.05. Scale bar = 100 μm

Additional staining for the predominant collagen in fibrocartilage, namely type I collagen, demonstrated a regional orientation and characteristic densely networked crimping pattern with an antero-posterior alignment in the intermediate (I) region in both species (Fig. 4A and B). Although the fiber orientation of type I collagen was similar between species, the percent area staining for collagen I was greater in the pig compared to sheep in the immediate posterior/intermediate, intermediate/anterior, and anterior discal attachments (P/I and A/I, respectively) as confirmed through quantitative histomorphometry (Fig. 4C).

**Fig. 5:**
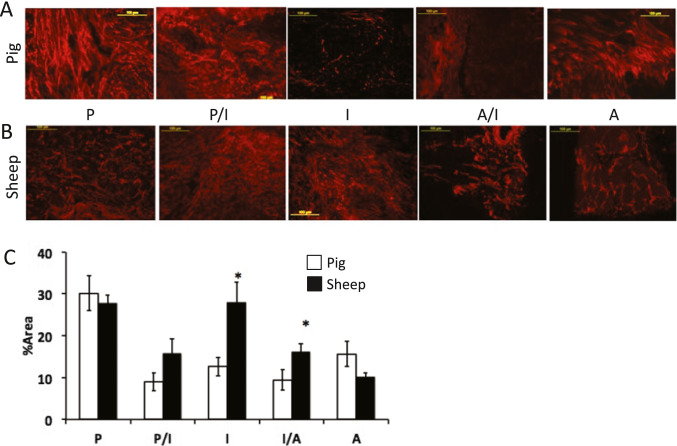
Immunohistochemistry for elastin of the **A** pig (*n* = 5) and **B** sheep (*n* = 5) TMJ disc-ligament complex demonstrates elastin organization that is region-specific. **C** Quantified elastin staining shows a greater percent area staining in the intermediate (I) articular disc region and the interface within the anterior-intermediate (I/A) discal attachment in the sheep compared to pig. Data represented as mean ± standard error of the mean (S.E.M.). Significant difference between *species, *p* < 0.05. Scale bar = 100 μm

Elastin fibers are found throughout the disc and disc periphery but occur at much lower levels than type I collagen irrespective of species. Elastin fibers were found to have similar orientation pattern as collagen but are less organized indicating a higher degree of branching and multidirectional orientation for both species (Fig. 5A and B). In porcine discs, the percent area staining for elastin was greatest in the posterior portion of the TMJ disc complex and relatively lower in all other regions (Fig. 5A and C). In sheep, both the posterior and intermediate zones showed highest proportion of the disc staining for elastin, thereby displaying a bimodal distribution pattern (Fig. 5B and C). The relative areas staining for elastin were significantly greater in the intermediate (I) and anterior-intermediate (A/I) regions of sheep than pig discs.

## Discussion

Large animal models are necessary to appropriately evaluate regenerative medicine strategies towards TMJ therapies [15]. It is of critical importance to characterize and elucidate the intrinsic similarities and differences between models, such as the pig and the sheep, to facilitate clinical translation to humans. Toward advancing these tissue engineering strategies for the TMJ, this study undertook the characterization and comparison of morphology and bone phenotype and disc ECM of sheep TMJ and that of often cited gold standard porcine model. One human sample was also used for gross morphologic and μCT data that together with historical information [16, 17] provides comparative insights of the relevance of each of these species for human applications.

Because joint and disc anatomy, biochemical composition, and biomechanical properties are determined by the mechanical function, chewing patterns and diet, the morphology and tissue phenotype of the TMJ reflects this structure–function relationship. Thus, despite the TMJ being a characteristic structure found in mammals, there is remarkable variation in morphology and function among species [18], such that no one animal model serves as an optimal analog for humans. Also, the large spectrum of TMJ disorders makes it impossible for any single model to recapitulate every type of TMJ dysfunction. Nevertheless, pig TMJs are similar in morphology to humans and other higher primates, and are also close to human in function, as the pig TMJ allows for a large range of motion including translational and rotational movements during chewing. For these reasons, the pig is considered the gold standard TMJ model [18]. Unlike the pig, the sheep TMJ joint anatomy permits a functional pattern specialized in translational movements in the transverse plane. Correspondingly, the sheep condyle is more concave than humans, although both sheep and pigs have similar TMJ disc size and shape [19]. Furthermore, our data, along with others [20–22], show that quantitative morphometry parameters of the trabecular condyle of the human also fall within the range found in pig and sheep TMJs. These findings suggest that while the overall shape of the sheep TMJ is more distinct from humans than that of pigs, both species serve as good models to assess bone mineral densities and other bone morphometry parameters.

Porcine and sheep TMJs have anatomical similarities in their discal attachments, in that both species, as well as humans, have a posterior attachment superiorly to the temporal bone and inferiorly to the posterior condyle, and an anterior attachment superiorly to the eminence and inferiorly to the anterior condyle [23, 24]. The histological and immunohistology evaluations of the disc and attachments demonstrate several similar characteristics in content and regional organization between pig and sheep, with both species showing the presence of collagen I and elastin and much of the matrix is a continuous blended network. However, we and others [25, 26] have shown complex regional variation in the matrix of the TMJ disc that may differ between species. In this work, while both species show low collagen I staining in the anterior region, porcine discs have significantly greater relative areas of the intermediate/posterior and intermediate/anterior zones staining for collagen than those of sheep. These differences and those of other ECM findings [25, 26] likely reflect disparities in functional and biomechanical demands placed on the disc in the two species.

Cross-linked elastin fibers are found in much lower quantities than collagen fibers which are 90% tissue mass [27, 28] with elastin fibers comprising only 1–2% of the tissue distributed throughout the disc and discal attachments. Yet, elastin fibers are critical in function since their compliant characteristics are responsible for restoring the original shape of the disc following loading [29–31]. The disc itself is highly fibrous and shows circumferential alignment of collagen fibers throughout the periphery and antero-posterior alignment through the central region (Fig. 4). This alignment of collagen fibers corresponds with the zonal structure–function relationship of the disc, with an antero-posterior alignment supporting the tensile forces imposed on the disc during functional movement. As shown here, the differences between elastin and collagen content of the attachments and the disc are relatively small between sheep and pig. However, even minor variations in ECM content and organization likely have a significant influence on the mechanical and functional properties of the tissue [32–34].

While absent from porcine discs, GAGs are localized in the intermediate and posterior/intermediate zones of sheep discal tissues (Fig. 3). These results corroborate those of others [26] and support the concept that GAG content is often high in joint soft tissues that sustain large compressive forces. In contrast to the lack of GAG localization in the porcine disc, humans TMJ discs demonstrate positive GAG staining [26], which corresponds with our findings in sheep in the intermediate and immediate posterior disc (Fig. 2), suggesting that sheep TMJ disc may be a more suitable human-analog for regenerating discal tissues with appropriate compressive tissue phenotype. A thorough overview of species and region-dependent biochemical properties suggest that in several species, including goats and humans, the intermediate zone has a higher GAG content than the rest of disc [25, 26], and concurs with our findings on the sheep. Furthermore, the posterior of the disc also has more collagen content than the other regions for all species. Besides these characterizations, it is notable that this current work is the first to also compare the bone morphology between human TMJs and pig and sheep.

Despite the utilization of one human TMJ for morphological and µCT data, the abundant historical findings on humans [16, 17] provide the necessary comparative information for our studies. While our findings from skeletally mature-aged sheep and pig add to the literature, it is likely that functional and maturational adaptations throughout the lifespan are additional important considerations in selection of animal models for TMJ regenerative therapeutics. Thus, age-related characterization of the TMJ in these large animal models would also be worthy of deeper analyses for further determining their applicability to human disorders and therapies. Lastly, quantitative biochemical measures need to be performed in the future to validate and provide further detail of our histological findings. Overall, our results suggest that while there are differences in the porcine and sheep TMJ morphologies, and bone and disc phenotypes, the sheep is as appropriate a model for TMJ regenerative therapies as the pig and each should be used for defined purposes to address specific concepts that closely mimic those found in humans.

## Conclusions

State-of-the-art regenerative strategies and evaluation require complementary in vivo biological and mechanical evaluation of the joint since these attributes of the TMJ environment together influence physiologic function and dysfunction. Our data suggest that depending on the specific research question or study, pigs and sheep may be suitable animal models for tissue engineering strategies of the TMJ depending on the precise engineering design, and functional and biological parameters being tested for applications to therapies in humans. Thus, further characterization of the TMJ in these large animal models would be highly valuable in determining the suitability of either for specific applications to human disorders and treatments.
